# Multicast reliable traffic engineering technique for SDN-Fog based IoUT

**DOI:** 10.1038/s41598-025-93383-w

**Published:** 2025-03-17

**Authors:** Reza Mohammadi

**Affiliations:** Computer Engineering Department, Faculty of Engineering, Bu-Ali Sina University, Hamedan, Iran

**Keywords:** Internet of underwater things, Software defined networking, Fog computing, Traffic engineering, Ocean sciences, Environmental impact

## Abstract

Underwater Internet of Things (IoUT) networks have gained considerable attention in recent years due to their wide-ranging applications in exploring and monitoring underwater environments, such as oceans and seas. Compared to previous decades, these networks have significantly improved our ability to understand and interact with aquatic ecosystems. However, several challenges remain, including the dynamic and variable conditions of underwater environments, high propagation delays, and the inherent unreliability of underwater communication channels. Ensuring reliable communication between underwater devices and surface nodes continues to be a critical concern. In this paper, we propose a novel solution that integrates Software-Defined Networking (SDN) architecture with Fog Computing to enhance communication reliability in underwater environments. The proposed method is based on an Integer Linear Programming (ILP) model designed to optimize underwater communication. Specifically, the approach considers communication between traffic-generating underwater nodes and surface-level nodes as a multicast operation. By solving this model, an optimized routing tree is generated that minimizes delays while maximizing reliability. The resulting routing structure is then disseminated to the underwater nodes by the network controller. Simulation results confirm that the adoption of an SDN-Fog-based architecture, combined with multicast routing, significantly enhances underwater communication performance, particularly in terms of reliability and network lifetime.

## Introduction

Underwater Internet of Things (IoUT) networks represent a rapidly evolving field, leveraging the capabilities of Internet of Things (IoT) technologies to explore and monitor aquatic environments, including oceans, seas, and other water bodies^1,2^. IoUT networks have revolutionized underwater applications such as environmental monitoring, resource exploration, disaster prediction, and military surveillance^3^. By deploying interconnected sensors and devices underwater, IoUT facilitates real-time data acquisition and analysis, enabling a deeper understanding of underwater ecosystems and phenomena.

Despite their vast potential, IoUT networks face several unique challenges. The underwater environment is inherently dynamic and variable, characterized by factors such as water currents, salinity, temperature variations, and interference^4^. Communication in such environments is further complicated by high propagation delays, low bandwidth, and the unreliability of underwater acoustic channels, which are prone to high bit error rates and packet losses^5^. These challenges make establishing reliable, efficient, and low-latency communication between underwater nodes and surface-level nodes a critical research problem.

Software-Defined Networking (SDN) offers a promising paradigm to address many of the challenges faced by IoUT networks^6^. By decoupling the control and data planes, SDN provides a centralized and programmable network architecture that enhances flexibility and scalability. In the context of IoUT, SDN facilitates dynamic routing, efficient resource management, and real-time network reconfiguration to adapt to changing underwater conditions^7^. The SDN controller, as the network’s central intelligence, can dynamically optimize communication paths and respond to disruptions, significantly improving the reliability and performance of underwater networks.

Fog Computing complements SDN by bringing computation, storage, and network resources closer to the IoUT nodes, reducing latency and dependence on centralized cloud infrastructures^8^. Unlike traditional cloud-based architectures, Fog Computing enables distributed processing and decision-making at the edge of the network. For IoUT, this is particularly beneficial, as it reduces the communication delays associated with sending data to distant cloud servers and enhances the overall Quality of Service (QoS) by processing critical data closer to its source.

In this paper, we propose a novel solution that integrates SDN architecture with Fog Computing to tackle the challenges of reliable communication in IoUT networks. Our method employs an Integer Linear Programming (ILP) model to optimize underwater communication by considering traffic between underwater nodes and surface-level nodes as a multicast operation. The model generates an optimized routing tree that minimizes delays and maximizes reliability. This optimized routing structure is then disseminated to underwater nodes by the SDN controller, ensuring efficient communication under dynamic underwater conditions.

The rest of this paper is organized as follows: Section 2 discusses the related work and highlights the limitations of existing approaches. Section 3 presents the proposed methodology, detailing the integration of SDN and Fog Computing with multicast routing. Section 4 provides the simulation setup and discusses the results. Finally, Section 5 concludes the paper.

## Related work

Given the novelty of IoUT networks, limited research has been conducted on the application of SDN architecture and multicast routing in this domain. This section aims to review some of the related studies in this area. Multicast communication has been recognized as an efficient approach for data dissemination in IoUT networks, particularly for applications requiring simultaneous data delivery to multiple nodes^9^. Protocols like PIM-SM^10^ and ODMRP^11^ have been adapted to IP or wireless mobile networks to support multicast operations. However, these protocols generally assume static network conditions, which limits their performance in dynamic underwater environments. Azizi and Zolanvari^12^ proposed a hybrid approach combining multi-cast routing and clustering to enhance QoS in underwater sensor networks. In their proposed method, after the formation of clusters, the underwater nodes connect to the cluster heads and transmit data to them. Although they employ intelligent approaches such as fuzzy logic, aspects such as the integration of SDN with Fog Computing have not been considered.

Zhang et al.^13^ introduced a novel scheduling strategy, based on TDMA (Time Division Multiple Access), for multicast communication in UASNs. Their key contributions include determining an upper bound on the throughput of multicast networks, where each packet has an equal number of intended destinations, by utilizing large propagation delays and investigating network topologies capable of achieving this bound. Their proposed method has successfully improved throughput, packet loss rate, and to some extent, the energy issue. However, their approach relies on complex mathematical computations and may not be applicable to many underwater topologies. Ze et al.^14^ presented a protocol named LBRP (Load Balanced Routing Protocol), to enable efficient multimedia data transfer in underwater sensor network scenarios. LBRP utilizes network stratification, delivering multimedia data packets sequentially, block by block and layer by layer. Simulation results indicate that LBRP enhances network lifetime and reduces multimedia data delay in underwater sensor networks. However, LBRP does not leverage the advantages of SDN and Fog Computing.

Shi et al.^7^ proposed an adaptive routing protocol called SQAR, designed for the Internet of Underwater Things (IoUT), which leverages reinforcement learning algorithms. SQAR intelligently selects routes to satisfy QoS requirements within the IoUT, promoting load balancing and reducing response times. Simulations showed that SQAR improves load balancing efficiency among SDN controllers and reduces QoS violations. However, SQAR does not encompass issues related to multicast communication and Fog computing. Click et al.^15^ proposed a framework based on SDN and NFV principles to provide services such as QoS assurance, fault recovery, resource management, and routing. Although the framework is broad in scope and utilizes SDN, it has not been fully implemented and does not support multicast communication. Li et al.^16^ introduced the SDN-QLTR routing approach for underwater sensor networks, emphasizing secure and trust-based routing. SDN-QLTR employs Q-learning algorithms for route computation. Simulation results show that SDN-QLTR improves security and various metrics such as delay, lifetime, and reliability. However, despite using SDN architecture, SDN-QLTR reliance on machine learning techniques demands substantial computational resources and energy for implementation in the controller, which may not always be feasible. Moreover, SDN-QLTR similar LBRP and SQAR, does not leverage the advantages of SDN and Fog Computing.

While existing studies have made strides in addressing underwater communication challenges, few have explored the intersection of SDN, Fog Computing, and multicast traffic engineering in IoUT. This gap highlights the need for solutions that optimize QoS in dynamic underwater environments through an integrated SDN-Fog-based approach. The proposed work addresses this gap by introducing a multicast traffic engineering technique that combines the strengths of SDN and Fog Computing to enhance underwater communication reliability and performance.

## Proposed architecture

In this section, the functionalities of the nodes in the proposed architecture are first presented, followed by an explanation of the process for calculating and announcing paths.

### Nodes functionalities

**Fig. 1:**
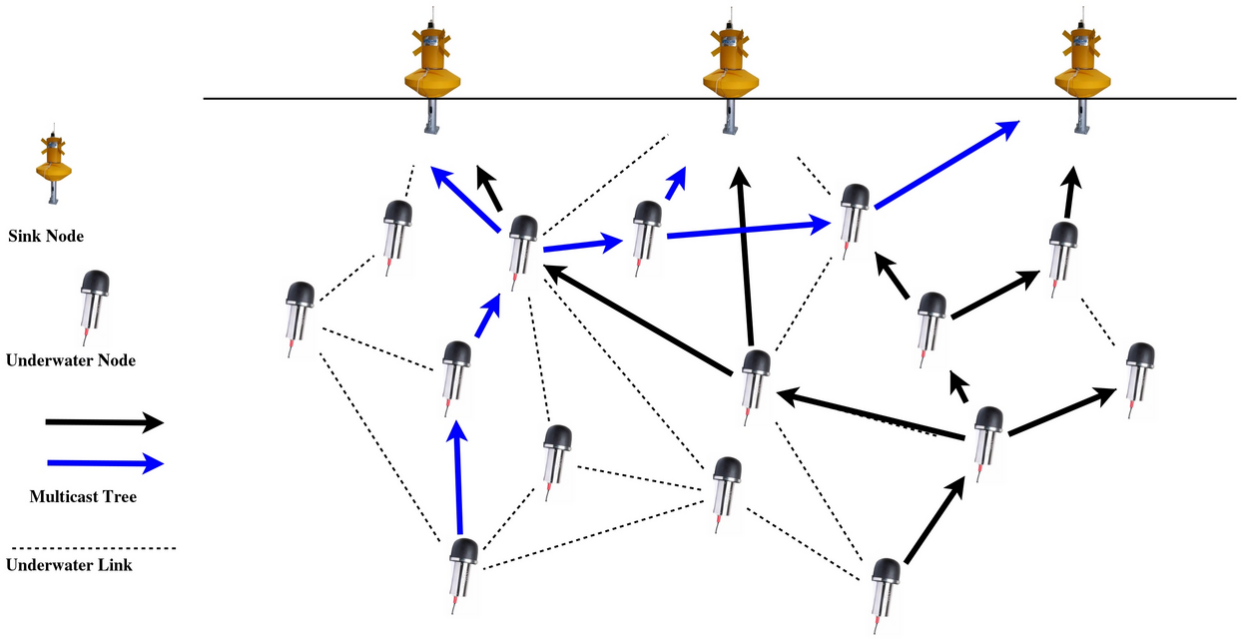
Proposed Architecture

In our proposed method, as depicted in Fig. 1, there are different nodes with different functionalities which are described as follows:

- *Sink Node:*These nodes are positioned at the water surface and are responsible for collecting data from underwater nodes. Given their proximity to underwater nodes, they can be considered as Fog nodes. After gathering data, they perform aggregation, summarization, and, if necessary, preprocessing before transmitting the data to the shore station. Essentially, the nodes deployed at the water surface implement the Fog layer in the proposed architecture. These nodes are equipped with two types of transceivers: an acoustic transceiver for communication with underwater nodes using acoustic signals, and a radio transceiver for communication with onshore node using electromagnetic signals. This ensures that the surface-level nodes can operate continuously without the energy constraints typically faced by underwater nodes, thereby enhancing the reliability and sustainability of the network. It is important to note that, since the proposed architecture also incorporates Software-Defined Networking (SDN), one of the sink nodes, in addition to the aforementioned tasks, also assumes the role of the network controller. Since these nodes are located at the water surface, they are equipped with solar-powered batteries, eliminating concerns related to energy limitations or the need for battery replacement.
- *Underwater Node:* These nodes are equipped with various sensors to monitor underwater phenomena. After collecting environmental data, the data is transmitted hop by hop toward the sink nodes located on the water surface. Additionally, these nodes are capable of forwarding data packets toward the sink nodes. The routing and packet-forwarding mechanisms are periodically communicated to these nodes by the controller node, which is one of the nodes deployed at the water surface.
- *Onshore Node:* This node is stationed on the shore and is responsible for transmitting the data received from the sink nodes to the central monitoring station via wired or wireless connections. The data is then processed and analyzed at the central station.

The proposed architecture ensures efficient data collection, processing, and transmission in underwater environments.

### Multicast path formulation

As mentioned in Section 1, the proposed architecture leverages the combined advantages of both SDN (Software-Defined Networking) and Fog computing. Since the underwater environment poses significant challenges and limitations for data transmission, sending data from transmitting nodes to the sink nodes located at the water surface may not always be successful. This is due to the fact that the underwater environment is characterized by extremely limited bandwidth and a high bit error rate. Additionally, the delay in underwater links is substantial, as long-distance communication in this environment relies solely on acoustic signals, which propagate at a speed of approximately 1500 meters per second. To enhance reliability and ensure that data reaches the surface-level nodes with a high probability, it is preferable to use multicast communication instead of unicast communication. In other words, rather than sending a packet to only one sink node, it is transmitted to multiple sink nodes. This approach ensures that there is always a high likelihood of the packet being received by at least one of the sink nodes. Given that, in the proposed architecture, the nodes at the water surface also function as Fog nodes, transmitting packets to them via multicast for preprocessing and aggregation ensures a highly reliable data delivery mechanism. By adopting this method, the proposed architecture not only addresses the inherent challenges of underwater communication but also enhances the overall reliability and efficiency of data transmission in such a constrained environment. In the proposed method, an optimal multicast routing tree is computed to implement multicast communication. Given that acoustic communication in underwater environments incurs significant delays, the cost of each underwater link can be considered equivalent to its Euclidean distance. In this paper, we formulate the multicast tree as the following Integer Linear Programming (ILP).

1 $$\begin{aligned} Objective=min \sum _{t\in T}\sum _{(a,b)\in G} {d}_{ab}x^t_{ab} ,\qquad {d}_{ab}=\frac{E_{ab}}{1500} \end{aligned}$$

                                    s:t: $${\forall a \in V}$$

2 $$\begin{aligned} & \sum _{b|(a,b)\in G} x^t_{ab} -\sum _{b|(a,b)\in G} x^t_{ab}=1 ,\qquad if a=s, t \in T \end{aligned}$$

3 $$\begin{aligned} & \sum _{b|(a,b)\in G} x^t_{ab} -\sum _{b|(a,b)\in G} x^t_{ab}=-1 ,\qquad if a\in D , t \in T \end{aligned}$$

4 $$\begin{aligned} & \sum _{b|(a,b)\in G} x^t_{ab} -\sum _{b|(a,b)\in G} x^t_{ab}=0 ,\qquad Otherwise \end{aligned}$$

5 $$\begin{aligned} & \sum _{(a,b)\in P_{sw}} {d}_{ab}x^t_{ab} \le \Delta ,\qquad w \in D, P_{sw} \in t \end{aligned}$$

6 $$\begin{aligned} & x_{ab}=0 \qquad or \qquad 1 , \qquad \forall (a,b) \in E \end{aligned}$$

where *G* is the network graph, $E_{ab}$ is the Euclidean distance between the nodes in 3D environment, *1500* is the speed of sound in underwater, *D* is the set of destination nodes, *s* is the source node, *T* is the multicast tree and $\Delta$ is the delay threshold. In the above formulation, the objective function minimizes the delay of the multicast tree. Equations 2 to 4 ensure the continuity of the paths between the source and all destinations. Constraint 5 guarantees that the delay of each path in the tree, from the source node to any destination, always remains below the threshold $\Delta$. Since underwater environments are characterized by significant delays, in this paper, Constraint 5 has been introduced with the aim of ensuring that the proposed method is applicable in delay-sensitive applications where the end-to-end delay must remain below a specified threshold. This constraint allows the proposed approach to be effectively utilized in scenarios where maintaining a bounded delay is critical, such as real-time monitoring or time-sensitive underwater communication systems.

### Multicast path calculation

As mentioned in Section 3.1, to leverage the advantages of SDN architecture, one of the sink nodes deployed at the water surface always acts as the network controller. This node is responsible for collecting information about the coordinates and energy levels of the nodes. To achieve this, the controller periodically sends a broadcast message to all underwater nodes at predefined intervals, requesting their current coordinates and remaining energy levels. In response to this message, all underwater nodes report their current coordinates and remaining battery energy to the controller. Naturally, nodes that have depleted their energy (i.e., their energy level has reached zero) cannot send any messages to the controller. As a result, the controller always maintains up-to-date information about the live nodes in the network.

By gathering all these messages, the controller constructs the network graph and formulates the ILP model described in Section 3.2. Since the aforementioned ILP model is NP-hard, solving it using mathematical methods, especially for environments with a large number of nodes, would be highly time-consuming. Therefore, in the proposed method, we employ a metaheuristic algorithm called TLBO (Teaching-Learning-Based Optimization) to solve the model, enabling the computation of the multicast routing tree in a significantly shorter time. Once the routing tree is computed, the controller communicates the calculated paths to the relevant nodes and instructs them to forward their packets according to the specified routes as depicted in Fig. 1. Given that the destinations in the multicast routing tree are the Fog nodes located at the water surface, the likelihood of packets successfully reaching the surface is significantly increased.

Since underwater nodes are constantly moving and not stationary, the controller periodically repeats the aforementioned process to maintain accurate information about the nodes’ locations and remaining energy levels. This ensures that the network remains adaptive to dynamic changes in the underwater environment, thereby enhancing the reliability and efficiency of data transmission.

Overall, the proposed architecture effectively integrates SDN with multicast routing to address the inherent challenges of underwater communication. By leveraging a centralized controller for dynamic topology updates and optimized path selection, the system ensures adaptive and energy-efficient data transmission. The periodic re-evaluation of node states further enhances reliability, making the approach well-suited for real-time and delay-sensitive underwater applications. These design choices collectively improve network performance while maintaining scalability and robustness in dynamic underwater environments.

## Performance Evaluation

This section presents a comprehensive simulation study to evaluate the performance of the proposed method. Given the absence of existing simulators that fully support all aspects of SDN-based IoUT communications, we developed a novel simulator. This simulator models underwater communication using acoustic signals and terrestrial communication using wireless radio signals while incorporating SDN architecture. The simulator was implemented using the Python programming language and leveraged several libraries, including NetworkX^17^ and Threading^18^. In evaluating our proposed SDN-based multicast routing method, we could not find a similar study applying the multicast routing in SND with the optimization techniques. To ensure a meaningful evaluation, we conducted a comparative analysis against a widely adopted hop-count-based mechanism, which serves as a standard benchmark in underwater communication. The hop-count metric is a widely adopted standard in computer networks and is employed by many established routing protocols^19^. This approach allows us to demonstrate the advantages of our method in terms of efficiency, reliability, and network performance. To ensure a fair comparison, the proposed method and benchmark approach were evaluated across various scenarios. All simulations were conducted on a computer equipped with an Intel Core i7-5930k-3.5 GHz CPU and 16 GB of memory. In all simulated scenarios, it was assumed that 10% of the nodes acted as traffic-generating nodes, with their locations fixed on the seabed. Additionally, the simulations included three fog nodes positioned at the sea surface, one of which served as both the controller and a standard sink node. The controller/sink node collected transmitted packets from underwater devices and simultaneously functioned as the SDN controller. The controller/sink was strategically positioned at the center of the topology on the water surface, while underwater devices were randomly deployed within a three-dimensional underwater area measuring $1000 \, \text {m} \times 1000 \, \text {m} \times 1000 \, \text {m}$. Each underwater device periodically announced its coordinates to the sink at 10-second intervals. The controller processed this information, computed optimal multicast tree, and communicated the results to the underwater devices every 10 seconds. Traffic-generating nodes produced traffic based on a Poisson statistical distribution, a commonly used model in network simulations^20^. This approach assumes that packets are generated independently at a constant average rate, with actual packet arrivals following a Poisson process. The simulation parameters are detailed in Table 1. The simulation parameters used in this study were carefully selected based on a combination of standard values from underwater communication research, relevant literature, and practical deployment considerations. For instance, the acoustic signal speed (1500 m/s) and communication range (300 meters) are widely accepted in underwater networks. The bandwidth (10 kbps) and transmission frequency (25 kHz) align with the characteristics of underwater acoustic channels. Additionally, the initial energy and power consumption values were chosen based on energy-efficient underwater communication strategies. Most of these values are taken from the LinkQuest^21^ underwater modem specifications, which is one of the most popular underwater modems. These parameters collectively ensure a realistic and practical evaluation of the proposed SDN-based multicast routing approach.

**Table 1:** Simulation Parameters

Parameters	Value
Initial Energy	10000 joule
Sink to onshore station distance	2 Km
Sink to onshore station Communication	IEEE 802.11/b (DBPSK)
Number of Underwater Sensors	50
Communication Range	300 meter
Simulation Area	1000*1000*1000 (3D)
Simulation Time	300 Seconds
Broadcast Packet size	60 byte
Acoustic signal speed	1500 meters/sec
Frequency	25 KHz
Tx power consumption	0.02 mWatt
Rx power consumption	0.01 mWatt
Bandwidth	10 kbps
Data Packet Size	100, 150,200 bytes
Number of Fog/Sink nodes	3
$\Delta$ Threshold	1000 meters( $\frac{1000}{1500}=0.66 sec$)
Packet Rate	5 to 25 packets/sec

### Simulation results analysis

In this section, the results are evaluated based on the following performance metrics.

**Fig. 2:**
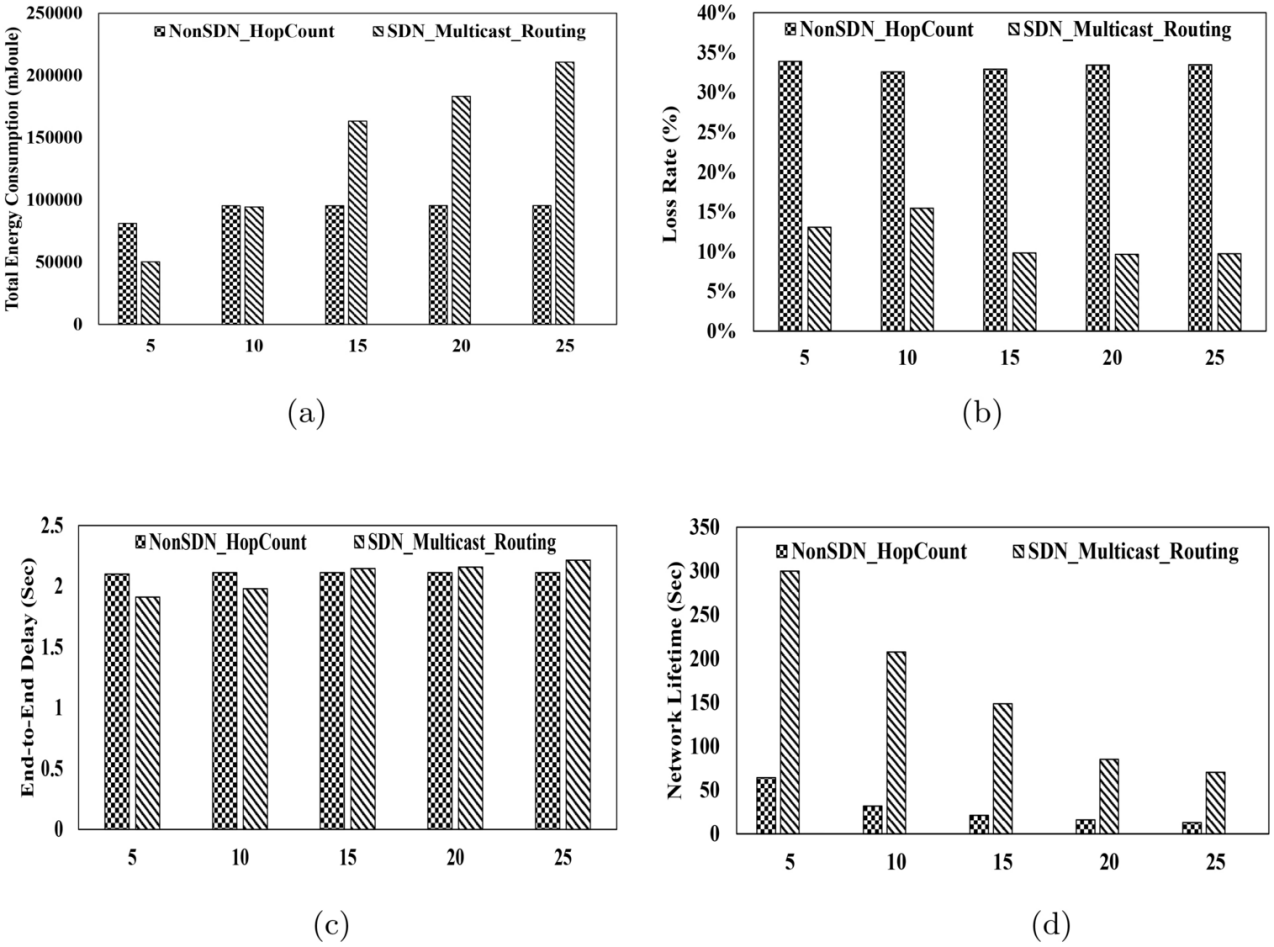
Performance Metrics for Packet Size 100 bytes. (**a**) Total Energy Consumption vs. Packet Rate, (**b**) Packet Loss Ratio vs. Packet Rate, (**c**) End-to-End Delay vs. Packet Rate, (**d**) Lifetime vs. Packet Rate.

**Fig. 3:**
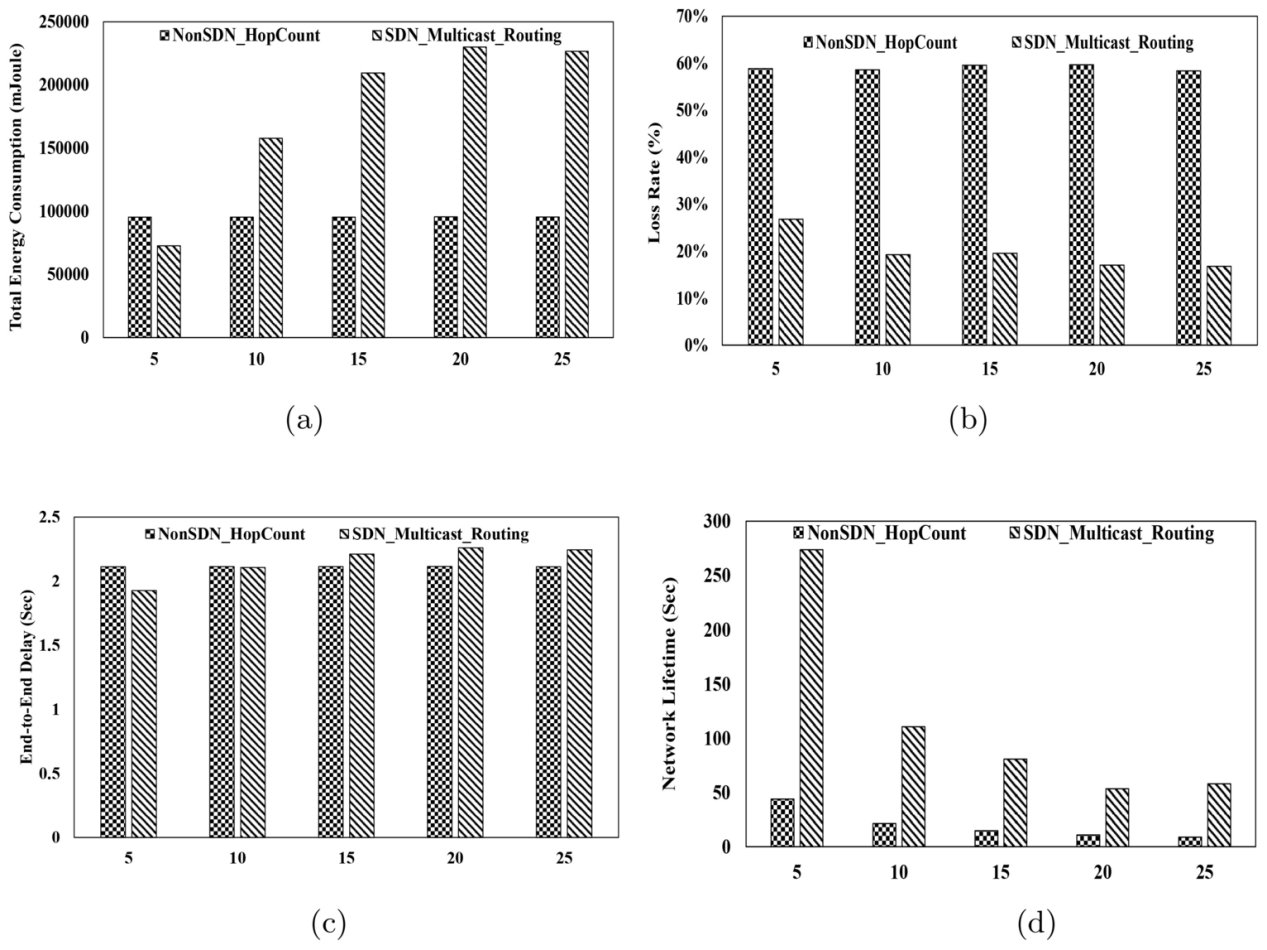
Performance Metrics for Packet Size 150 bytes. (**a**) Total Energy Consumption vs. Packet Rate, (**b**) Packet Loss Ratio vs. Packet Rate, (**c**) End-to-End Delay vs. Packet Rate, (**d**) Lifetime vs. Packet Rate.

**Fig. 4:**
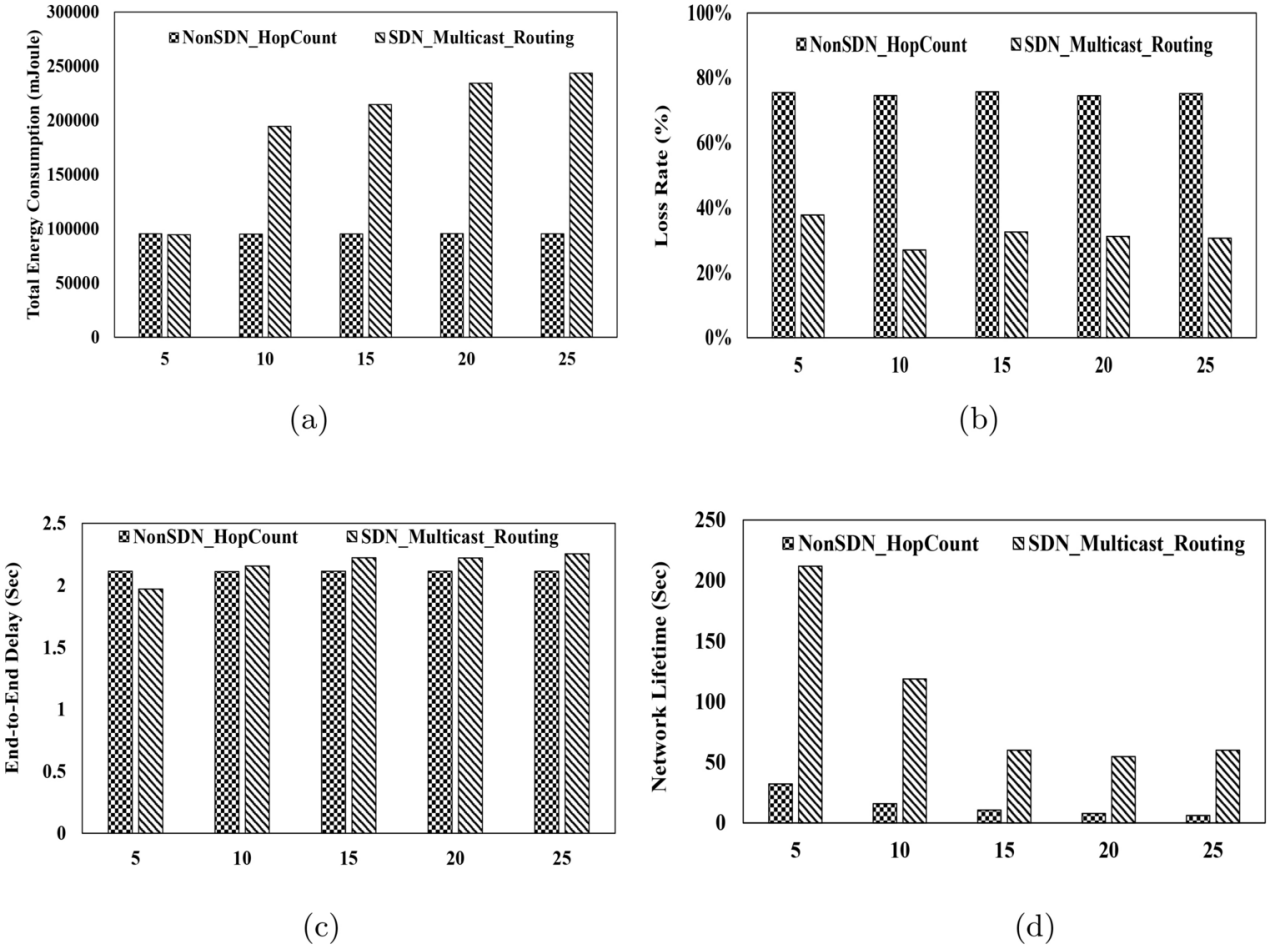
Performance Metrics for Packet Size 200 bytes. (**a**) Total Energy Consumption vs. Packet Rate, (**b**) Packet Loss Ratio vs. Packet Rate, (**c**) End-to-End Delay vs. Packet Rate, (**d**) Lifetime vs. Packet Rate.

#### Total energy consumption

This metric refers to the total energy consumed in the scenario by all nodes, including underwater nodes, sink nodes, and the controller. It is expressed in millijoules (mJ) in this study. As illustrated in Fig.2.a, Fig.3.a and Fig.4.a, energy consumption in the multicast routing method is consistently higher than that of the hop-count-based routing method across various packet transmission rates and packet sizes. Moreover, as the transmission rate and packet size increase, the difference in energy consumption becomes even more pronounced. The primary reason for this higher energy consumption lies in the nature of multicast routing, where identical packets are intended to be delivered to multiple sink nodes. This process requires the establishment of multiple paths, and packets must traverse different routes to reach the respective sinks. Additionally, in the multicast routing tree, which consists of multiple branches, the packet originating from the source node may need to be forwarded to several branches. Consequently, multiple copies of the packet are created, each directed toward a specific sink. This duplication and distribution process inherently leads to higher energy consumption in the multicast routing method compared to the hop-count-based routing method. Furthermore, in the proposed multicast routing method, the use of the SDN architecture contributes to increased energy consumption. In this approach, underwater nodes periodically report their coordinates and remaining energy to the controller. The controller, in turn, computes the routing tree and communicates it back to the nodes. This process inherently requires additional energy, further contributing to the overall energy consumption in the system.

#### Packet loss rate

This metric represents the percentage of packets transmitted by the source nodes that are not received by any sink node at the destination. Fig.2.b, Fig.3.b and Fig.4.b show packet loss ratio for different packet rates and packet sizes. The results presented in these figures indicate that the packet loss rate in all scenarios is significantly lower in the proposed method compared to the hop-count-based method. As mentioned in the introduction, one of the primary objectives of the proposed method is to enhance reliability in underwater communications. To achieve this, instead of sending packets to a single sink, the proposed method delivers packets to multiple sinks through a routing tree. In this setup, even if some of the paths within the tree are disrupted for any reason, other paths are likely to remain available, enabling the packet to reach at least one sink. Consequently, the proposed method exhibits a substantially lower packet loss rate. In contrast, the hop-count-based method routes packets through a single path to a single sink. If this path fails, the packets cannot reach the sink node located on the water surface, resulting in a higher packet loss rate.

#### Average End-to-End delay

This metric measures the time it takes for a packet to travel from the source node to one of the sink nodes. As illustrated in Fig.2.c, Fig.3.c and Fig.4.c, the end-to-end delay in the proposed method and the hop-count-based method shows only a slight difference, with the hop-count-based method achieving marginally lower delays. This minor difference arises because the hop-count-based unicast routing method adopts a greedy approach to minimize the number of hops, always calculating the shortest path to the sink node. In contrast, the proposed multicast routing method, as described in section 3.2, computes the routing tree using an ILP model while considering delay constraints. This approach ensures that the resulting solution is a tree rather than a single path. Consequently, the tree may include multiple branches, and the end-to-end paths within the tree are not necessarily the shortest paths between source-destination pairs. Nevertheless, the results demonstrate that the proposed method does not exhibit significant delays and remains closely comparable to the hop-count-based method in terms of end-to-end delay.

#### Network lifetime

This metric is defined as the time until the last data packet is received by one of the sink nodes. Beyond this point, no data packets are received by any sink node. Fig.2.d, Fig.3.d and Fig.4.d show network lifetime for different packet rates and packet sizes. These figures clearly demonstrate that the network lifetime in the proposed method is significantly higher compared to the hop-count-based routing method. Although the proposed method consumes more energy, as indicated by the energy consumption results, it ensures a longer network lifetime. The primary reason for this advantage lies in the dynamic nature of the proposed method. When the controller collects the coordinates and remaining energy of the nodes, nodes with depleted energy do not send updates to the controller. Consequently, the controller continuously constructs the network graph based on the active nodes and calculates the optimal routing tree accordingly. Since this process is performed periodically, packets are never routed through paths containing nodes with zero energy. As a result, as long as there is a viable path between the source node and the sink nodes located on the water surface, packet exchange continues seamlessly. The network lifetime is considered to end when the controller can no longer compute a routing tree in the network graph. In contrast, the hop-count-based routing method exhibits significantly lower network lifetime. This is because it calculates a single path leading to a sink without accounting for the energy levels of the nodes. As a result, connectivity is lost quickly, and the network experiences an early termination of communication.

## Conclusions

In this study, we proposed a novel SDN-based multicast routing method for underwater communications within the IoUT. The proposed approach integrates Fog computing with the flexibility of SDN to dynamically construct optimal routing trees based on on-the-fly feedback from underwater nodes, including their coordinates and remaining energy levels. This adaptive mechanism improves network reliability and lifetime while addressing key challenges in underwater communication environments. Simulation results demonstrate that the proposed method significantly outperforms conventional hop-count-based routing in terms of network lifetime and packet loss rate. The use of multicast routing enables the delivery of packets to multiple sink nodes, ensuring communication reliability even in the presence of path failures. Although the proposed method incurs higher energy consumption compared to hop-count-based routing, it effectively balances this trade-off by extending the network lifetime. Additionally, the end-to-end delay of the proposed method remains comparable to that of hop-count-based routing, with only a slight difference attributable to the use of a routing tree rather than a single shortest path.

## Data Availability

The datasets used and/or analysed during the current study are available from the corresponding author on reasonable request.
